# Inactive but awake behaviour as indicating a depression-like state in mice: aetiological factors and association with adult hippocampal neurogenesis

**DOI:** 10.1098/rsos.251069

**Published:** 2025-10-15

**Authors:** Anna Trevarthen, Ruth Davy, Emily Finnegan, Rebecca-Leigh Railton, Elizabeth Paul, Michael Mendl, Tom Smulders, Carole Fureix

**Affiliations:** ^1^ Bristol Veterinary School, University of Bristol, Bristol, UK; ^2^ School of Psychology, Newcastle University, Newcastle upon Tyne, UK

**Keywords:** inactivity, mouse, hippocampus, neurogenesis, dentate gyrus, behaviour

## Abstract

In laboratory mice, ‘inactive but awake’ (IBA) home-cage behaviour involves animals being spontaneously motionless with eyes open, not interacting with their surroundings. Conventional (barren) housing typically triggers IBA more than comparatively enriched environments. Compellingly greater IBA is associated with some depression-like features in mice and we further explored this through three aims. First, we aimed to replicate previous results highlighting environmental and genetic (using two strains of mice: DBA/2J and C57BL/6J) aetiological contributors to IBA. Second, we explored whether the performance of IBA varied as the level of enrichment was either increased or reduced. Third, we opportunistically investigated whether elevated IBA predicted lower density of immature neurons in the dorsal (dDG) or ventral dentate gyrus (vDG) of the hippocampus. As expected, mice housed in conventional cages displayed more IBA than those in comparatively enriched cages and even more so in DBA/2J mice. As predicted, enrichment loss generally increased IBA while enrichment gain decreased IBA. Unsurprisingly, immature neuron density was lower in conventional compared with enriched cages, although only for vDG. Elevated IBA predicted reduced immature neuron density in the dDG, and this effect tended to be stronger for C57BL/6Js. We discuss the result implications, study limitations and future research directions.

## 1. Introduction

In captivity, animal activity is commonly used as an indicator of welfare. Elevated levels of activity can be associated with some abnormal repetitive behaviours, such as pacing, bar-mouthing and spinning, which are often linked with poor welfare [1]. However, increased inactivity can also be associated with compromised welfare. Recent work has identified a specific form of inactive behaviour which appears to occur similarly across several captive species (e.g. mice [2,3]; mink [4,5]; macaques [6]; dogs [7,8]; horses [9]). Termed ‘inactive but awake’ (IBA) behaviour in mice, it has been documented in the home environment and involves animals being spontaneously motionless (but awake, i.e. with eyes open) while making no interaction with their surroundings. In laboratory mice, IBA is reliably displayed more often in conventional (more barren) cages than those that are bigger, better provisioned with enrichment and are more preferred/valued than conventional cages [10,11], indicating it may be associated with poorer welfare [2,3,12–15]. The ethological function of IBA remains unknown, although it may promote low activity and energy conservation when resources are lacking [16,17]. The type of affective state that accompanies IBA has not been extensively researched either; however, initial evidence indicates that IBA is associated with depression-like features in mice (reviewed in [14]).

Clinical depression in humans is a heterogeneous mood disorder with nine potential symptoms, at least five of which (including anhedonia, ‘loss of interest or pleasure’ or low mood) need to be experienced for at least two weeks for a diagnosis to be made ([18], P160). Compellingly, reduced activity and environmental engagement are two of the features of clinical depression in humans [18], giving translational support for IBA as a potential marker of a depression-like symptom in laboratory mice. Furthermore, elevated IBA may predict reduced preference for sucrose (a typical proxy for anhedonia—unpublished data), and elevated sleep and weight (unpublished data, [19]), which are all diagnostic criteria of human depression [18]. Moreover, recent work has found that IBA performance is alleviated by both environmental (enrichment) and pharmacological (antidepressant) manipulations that mimic curative factors in human depression [3].

In this article, we further explored the hypothesis that greater IBA behaviour is a depression-like feature and focused on three specific aims. First, we aimed to replicate previous results highlighting environmental and genetic aetiological factors of IBA behaviour. Following the diathesis-stress model of depression development in humans [20], and based on previous work in mice [2,3,13,15,21], we predicted that chronically barren housing would trigger more IBA. We housed mice in conventional ‘shoe box’ (small, relatively non-enriched (NE)) cages and compared them with those housed in larger, highly enriched (EE) cages. We used two strains of inbred mice, C57BL/6J and DBA/2J and, based on previous findings, expected them to differentially display IBA [2,15]. We also expected that such a strain-related difference would be exacerbated in NE cages. Such replication of previous results is important to strengthen the validity (e.g. [22]) of the measure of IBA.

Second, we explored whether the amount of time mice spent displaying IBA varied as the level of enrichment was manipulated. Some mice were transferred to a conventional ‘shoe-box’ cage from a highly EE cage, a change in living environment which has previously been shown to induce cognitive ‘pessimism’ (a proxy of negative emotional affective state) in rats [23], starlings [24] and pigs [25] and increases in IBA in mice [3]. We expected our mice to display an increase in IBA following this environmental adjustment. Conversely, other mice were transferred from a conventional cage to a larger highly EE home cage. We expected these mice to display a reduction in IBA performance following their housing manipulation, as also observed in [3]. Two further groups of mice remained in their initial housing (either conventional ‘shoe-box’ or highly enriched) for the duration of the experiment and experienced no environmental adjustment (acting as stable control groups).

Our third aim was to opportunistically investigate, for the first time, whether greater levels of IBA behaviour predicted lower density of immature neurons in the dorsal (dDG) or ventral dentate gyrus (vDG) regions of the mouse hippocampus. Hippocampal neurogenesis is reportedly reduced in some human patients with clinical depression [26–28], making it a potential biomarker for depression in animals. It has been well established in mice that reduced neurogenesis is displayed in the dentate gyrus (DG) in response to chronic stress procedures such as unpredictable chronic mild stress and chronic restraint (e.g. [29–31]), which are often used to model depressive-like states [32,33]. Some studies have identified differential densities and maturation timings of neurons across the ventral–dorsal axis of the DG in mice (e.g. [34,35]). Although the literature is not clear-cut [36], in the mammalian brain the vDG (termed anterior DG in primates) is thought to be involved in emotion dysregulation [37], whereas the dDG (termed posterior DG in primates) is thought to be involved in mnemonic functions (which can also be impacted in depression [18,38]), such as contextual learning and exploration [39].

Initial work in this field has found significantly reduced hippocampal volumes for C57BL/6 mice, which displayed high levels of IBA [19]. We therefore aimed to explore how neurogenesis in both poles of the dentate gyrus covaries with IBA levels and did so by measuring immature neuron density as a marker of the number of new neurons being generated [40]. We chose to model this using the stably housed treatment groups (and not in those exposed to changing living environments) because we were only able to take one measure of immature neuron density (i.e. on brain removal at the end of the experiment). The change in environmental conditions applied to the other two treatment groups meant that they experienced *both* EE and NE conditions, expected to cause opposite changes in levels of IBA throughout the experiment, and making it difficult to predict effects on end-of-life neuron density. However, using the stably housed groups, we were able to predict that mice housed in the comparatively NE environment for the duration of the study would display more IBA (thereby displaying a more depressive-like phenotype) and have reduced hippocampal neurogenesis and therefore lower densities of immature neurons when compared with highly EE mice.

## 2. Methods

### 2.1. Animals, housing and husbandry

**Figure 1.**
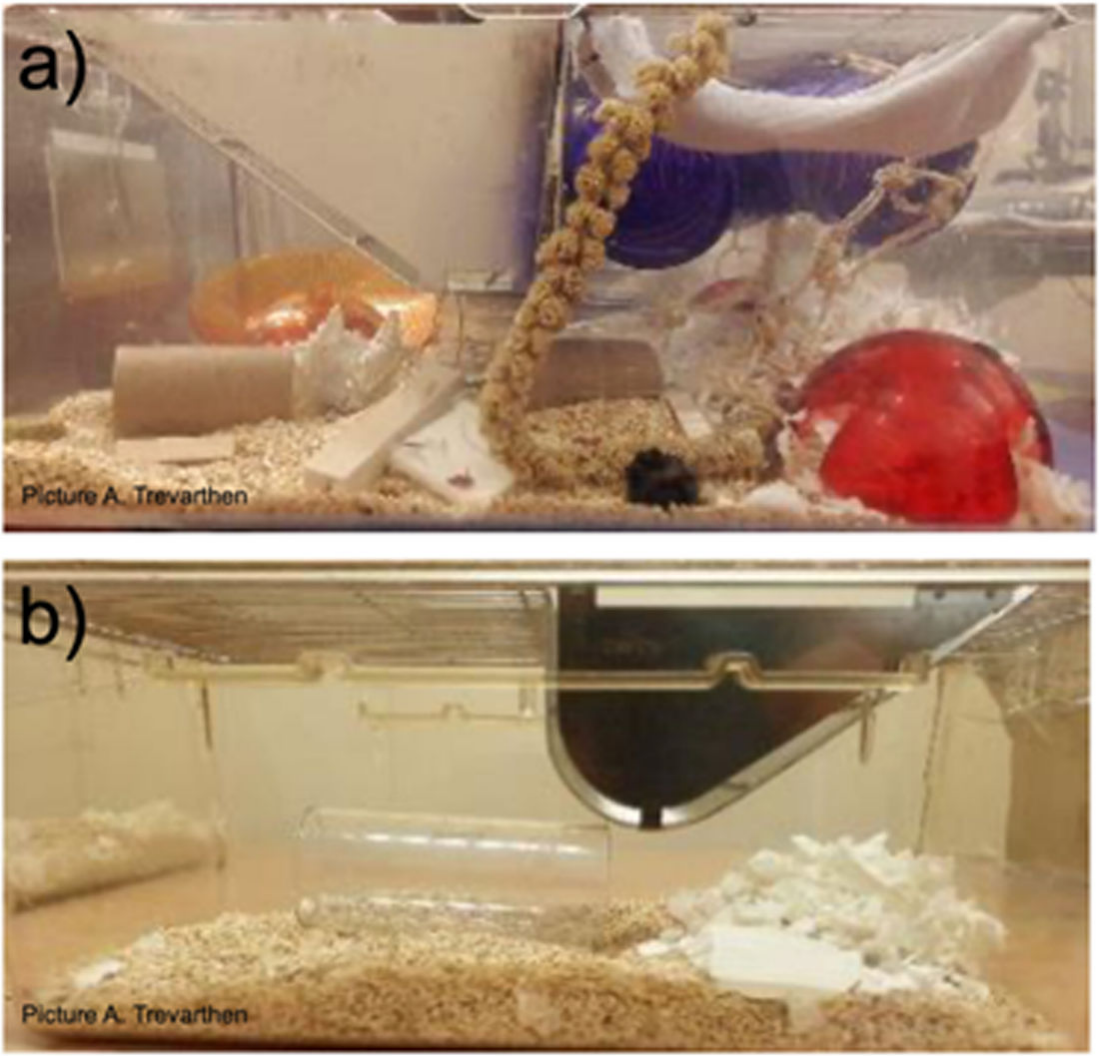
Photographs of an EE housing cage, EE (a) and a NE housing cage (b). Photographs not to the same scale (NE cages are smaller than EE cages). Photo credit: Anna Trevarthen.

Thirty-one female C57BL/6J (C57, hereafter) and 31 female DBA/2J (DBA, hereafter) mice (*Mus musculus*; Charles River, France) were pair-housed post-weaning in either highly EE (*n* = 15 cages) or NE (*n* = 16 cages) transparent Techniplast cages. Estimates of animal numbers were based on power analyses using data from [2] (power 80%, significance criterion two-tailed 0.05, Cohen’s *d* effect size 0.7 (minimum expected means difference 0.011 and estimated standard deviation 0.01562), replicated using several calculators). Upon arrival at the laboratory (at three–four weeks of age), each mouse was weighed, and one C57 (black) and one DBA (brown) mouse of similar weight (±2.5 g) were pseudo-randomly allocated to each cage (mouse selected at random from mice of similar weights and allocated to the cage with a weight-matched cage-mate). The mixed-strain housing enabled individual identification within pairs, removing the need for invasive marking procedures [41]. The EE cages were 44 cm L × 34 cm W × 20 cm H and contained sawdust (IPS), two handfuls of nesting material, a plastic transparent shelter (∅ 15 cm, 5.5 cm H, Biopac UK), a red igloo with a running wheel (fast-trac, Datesand), a transparent polycarbonate handling tunnel (13 cm L, ∅ 5 cm, Datesand), a flexible transparent plastic tunnel (∅ 6 cm, 30 cm L), which was attached to the cage lid, three aspen gnawing blocks (two small: 5 cm L × 1 cm W × 1 cm H, one large: 10 cm L × 2 cm W × 2 cm H, Datesand), a small pine cone, two nestlets (Ancare, USA), a hammock (approximately 12 × 12 cm) made from a sock attached to the lid, a sisal rope ladder and one half of a coconut shell (approximate dimensions: 12.7 cm L × 7.6 cm W × 5 cm H, Little Cherry Ltd), which was attached to the lid with sisal rope (figure 1a). A millet spray (Pets at Home) was attached to the lid of each cage and was refreshed during monthly cage cleaning. NE cages were 37 cm L × 21 cm W × 14 cm H and contained sawdust (IPS), one handful of nesting material, a small piece of cardboard (approximately 5 × 5 cm) for gnawing and a transparent polycarbonate handling tunnel (13 cm L, ∅ 5 cm, Datesand) (figure 1b). Food (LabDiet) and water were available ad libitum and animals were kept under a 12 h reversed light–dark cycle (lights on 1900−0700). All cages were housed within three Scantainers (Scanbur BK) in a pseudo-random order that ensured an equal spreading of experimental treatments between the three Scantainers and within shelves for each Scantainer. NE cages were cleaned every week and EE cages were cleaned every four weeks. Temperature (mean ± s.e.m, 20.9 ± 0.5°C) and relative humidity were controlled at the room level (Scantainers used as shelving units with doors open) and were checked daily.

Following three weeks of housing in their initial environment, half of the mice (*n* = 8 from EE cages and *n* = 8 from NE cages) were moved into the alternative environment (i.e. NE cages if initially housed in EE cages (group labelled: ‘EE-NE’); and EE cages if initially housed in NE cages, (group labelled ‘NE-EE’)), while all other mice stayed in their initial environment (*n* = 7 in stable-environment control group ‘EE-EE’ and *n* = 8 in stable control group ‘NE-NE’). All mice were transferred to a clean cage regardless of whether their environment was being altered. Mice then remained in this environment for the rest of the experiment. When being moved between environments (and if handled at all during the experimental period), all mice in NE cages were handled by the tail, while mice in EE cages were handled using their home-cage Perspex handling tunnel, following a validated method shown to reduce stress in laboratory mice [42–44].

### 2.2. Behavioural scan sampling

The behaviour relevant to the hypothesis under test was being IBA, defined after Fureix *et al*. [2] (see also [21]) as ‘mouse motionless, muzzle in sight and eyes open, for at least 15 s’. Behavioural observations of mice in their home cage began on day 4 after arrival in the laboratory, following three days of acclimatization. All observations were conducted during the animals’ dark (active) phase under red ambient light, for three weeks, four days per week, over four 90 min time blocks per day: 9.30−11.00, 11.30−13.00, 13.45−15.15 and 15.45−17.15. Behaviour was recorded through live scan sampling [45], switching from scan to 15 s focal sampling to allow for differentiation between behaviours characterized by a lack of movement (e.g. IBA versus sleeping) as in e.g. [2,7]. In total, 24 scan samples were taken per mouse each day, spread evenly across the four blocks (totalling 288 scan samples/mouse for this first phase of observation (IBA observation 1, pre-adjustment phase)). During the first week of observations, two experimenters (A.C.T., E.F.) were present during each scan to simultaneously record the behaviour of each mouse. Because inter-observer reliability analyses indicated a high degree of agreement (Cohen’s kappa value: 0.978), all subsequent scan samples were split between the two experimenters.

**Figure 2.**
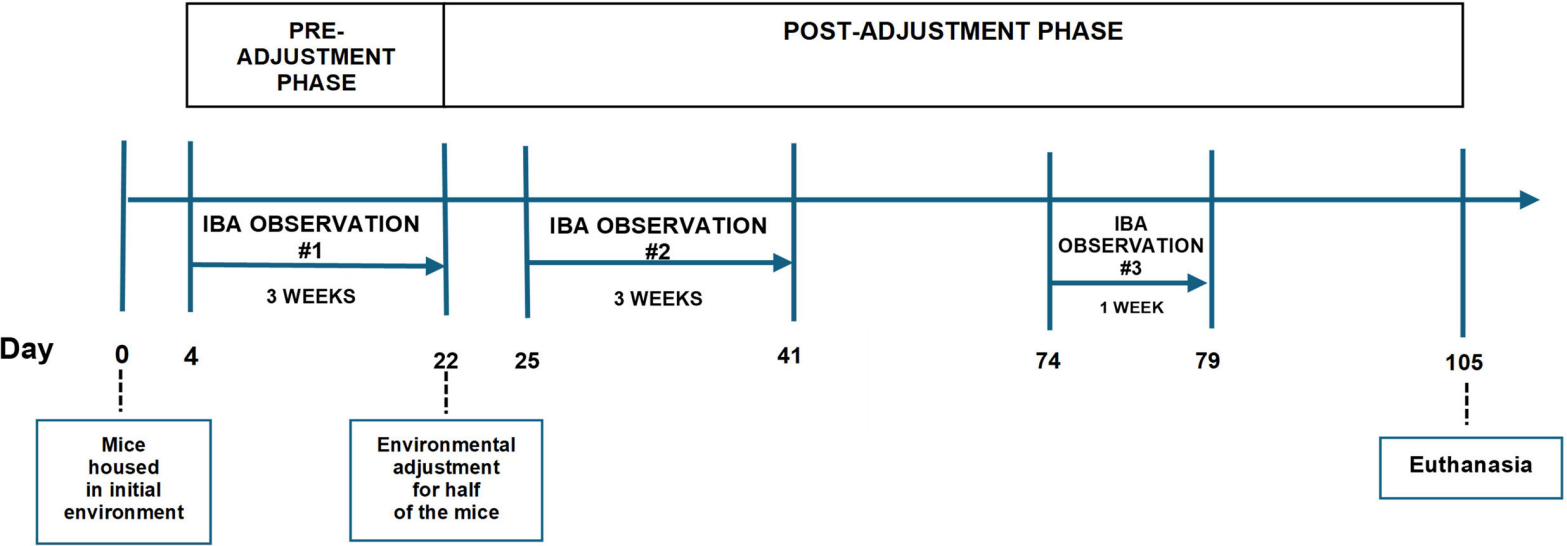
The experimental timeline shows the pre- and post-environmental adjustment phases of the experiment and the time points at which IBA behaviour was observed.

Environmental adjustment, i.e. moving into the alternative environment, or staying in the initial environment for control groups, happened on the day following the last day of observation in the pre-adjustment phase, with a naive experimenter pseudo-randomly switching the position of cages within each Scantainer to ensure the behavioural observers were blind to the environmental adjustment each cage had undergone (treatments remained equally spread between the three Scantainers and within shelves for each Scantainer). Home-cage behaviour was monitored again for three consecutive weeks, starting two days after environmental adjustment (IBA observation 2, post-adjustment phase), following the above-described procedure (with the exception that scan samples were immediately split between two experimenters) and also totalling 288 scan samples/mouse during this second phase of observation. Finally, we had the opportunity to observe IBA in the mice during the eighth week following environmental adjustment (IBA observation 3, post-adjustment phase). This followed the procedure outlined for observations 1 and 2 with the exception that behavioural observations took place over one week instead of three, totalling 96 scan samples/mouse during this observation. Since we have little information about the development of IBA over time, we took this one-week opportunity to gain this additional data. The experimental time points are outlined in figure 2.

### 2.3. Immunohistochemistry

This immunohistochemistry aim developed opportunistically throughout the experiment, so mice were euthanized once staff training in brain removal was completed. Following euthanasia, the brain of each mouse was immediately (within 15 min) extracted from the skull. The brains were fixed in 4% paraformaldehyde for 48 h and cryoprotected in 30% sucrose until they sank, before being embedded in optimal cutting temperature (OCT) compound. Samples were then frozen at −80°C until they were sent for analysis at Newcastle University (conducted by R.D. and T.S.). Sagittal sections were cut using a cryostat (Leica CM152, Leica Biosystems) at 40 μm thickness and stored in a cryoprotectant solution (0.01 M phosphate-buffered saline (PBS), 30% glycerol, 30% ethylene glycol) in four parallel series. Free-floating sections were stained for Doublecortin (DCX; a marker of immature neurons— [46]. For each brain, the sections were spaced by 160 μm, except for two brains, where they were separated by 120 μm for practical reasons. Sections were washed in 0.01M PBS at room temperature, and endogenous peroxidase was inhibited for 30 min in 1% H_2_O_2_ in dH_2_O. Tissue was washed three times in PBS and was then incubated for 1 h in blocking solution (0.01M PBS, 0.3% Triton X-100, 1% bovine serum albumin, 2% normal goat serum (NGS)). Samples were then incubated overnight (at least 16 h) at 4°C in rabbit polyclonal to DCX primary antibody (AbCam, ab18723; 1 : 1000) in 0.01M PBS with 0.3% Triton X-100. The following day, after washing, the samples were incubated for 120 min at room temperature in 1 : 500 biotinylated anti-rabbit IgG secondary antibody, made in goat (Vector Labs, BA-1000). Samples were washed before incubation in 1 : 250 Horseradish Streptavidin (Vector Labs, SA-5004) for 60 min. Following washes in PBS and dH_2_O, 30 s chromogen incubation in 3,3′-diaminobenzidine (DAB) was conducted by diluting SIGMAFAST tablets (Sigma-Aldrich, D4418) in pure water to a final concentration of 0.35 mg ml^−1^. Brain sections were then rinsed in water, and free-floating sections were mounted on slides by hand and cover-slipped using Eukitt (Sigma-Aldrich, 03989) before image analysis.

### 2.4. Image analysis

For each brain, images of the dDG (minimum three sections) and the vDG (minimum one section) were captured at 40× magnification using a Leica DMLB microscope connected to a video camera (Optronics Microfire Digital Camera, USA). Images were stitched together using GNU Image Manipulation Program 2.10 (USA) so a complete dDG or vDG section could be analysed. Cell count and length of DG were quantified in a blinded fashion using Fiji ImageJ 1.53 (National Institutes of Health, USA) by two separate assessors. A preliminary data quality assessment was conducted to analyse the correlation between cell count and DG length measurements collected by two independent, blinded assessors to evaluate the measure. The data from the two assessors were strongly correlated (Pearson correlation coefficient, *r* = 0.953, *p* < 0.001), so subsequent measurements were split between the assessors. Immature neuron density was calculated from cell count and DG length measures by first taking a sum of all the dDG counts and lengths and all the vDG counts and lengths, and then calculating the average immature neuron density of dorsal or ventral sections per brain by dividing total cell count by total cell length. Samples were successfully obtained for all mice except one in the EE-EE treatment group.

### 2.5. Statistical analyses

Data were analysed in R Studio version 4.2.1 using the lme4 package. We constructed generalized linear mixed models with random intercepts, but fixed slopes. Specific predictions are presented at the start of each aim in §3.

Aim 1 was to replicate previous results obtained in another laboratory [2,15] highlighting environmental and genetic aetiological factors of IBA. To investigate this aim, only data from the pre-environmental adjustment phase (i.e. IBA observation 1) were analysed, using the proportion of IBA behaviour shown by each individual mouse (while in view, to control for the increased likelihood that mice would be out of sight during scan sampling of EE cages) during scan sampling as the dependent variable. Within the model, ‘cage’ and ‘mouse’ were included as random effects. Treatment (i.e. EE cage, NE cage), strain (C57, DBA), and the two-way (treatment × strain) interaction were included as fixed-effects.

To investigate aim 2 (to explore whether the amount of time mice spent displaying IBA would increase with environmental removal, and conversely would decrease when removing environmental enrichment)*,* we constructed models calculating the *change* between observations in the proportion of IBA performed while visible. We used one model for the change in IBA between observations 1 and 2 (i.e. IBA observation 2 minus IBA observation 1) and another for the change in IBA between observations 1 and 3 (i.e. IBA observation 3 minus IBA observation 1). Within each model, ‘cage’ and ‘mouse’ were included as repeated (nested) random effects, treatment group (EE-EE, NE-NE, NE-EE or EE-NE), strain (C57, DBA) and the two-way (treatment × strain) interaction were included as main effects.

Our third aim was to opportunistically investigate whether a greater level of IBA predicts lower immature neuron density in the dDG or vDG. We chose to model this using only the stably housed treatment groups (i.e. NE-NE and EE-EE), because the reversed environmental adjustment applied to the other treatment groups (i.e. NE-EE and EE-NE) was predicted to inversely influence the measures during the pre- and post-environmental adjustment phases. As such, we could not make accurate predictions for these two groups about the relationship between immature neuron density (measured once at the end of life) and IBA, given the different life experiences of these mice. We conducted a first model to measure the effect of the region of the dentate gyrus on immature neuron density. Along with region (i.e. dDG or vDG), treatment (NE-NE or EE-EE), strain (C57 or DBA) and the two- and three-way interactions were included as fixed-effects. We then conducted two separate models using either the dDG immature neuron density or the vDG immature neuron density as the dependent variable. Separate models were necessary to avoid pseudo-replication of data by including IBA twice. Because we were only able to take one measure of immature neuron density (i.e. on brain removal at the end of the experiment), we used IBA observation 3 data taken on the eighth week following environmental adjustment (i.e. as close to brain removal as possible) as a predictor covariate in our models. Within each model (one per region of the hippocampus), ‘cage’ and ‘mouse’ were included as nested random effects. In addition to IBA observation 3, which was included as a covariate, treatment (EE-EE or NE-NE), strain (C57, DBA) and both the two- and three-way (IBA × treatment × strain) interactions were included as fixed effects.

For each of the models produced, the assumptions of parametric testing were checked using Shapiro–Wilk normality tests on the model residuals. Where necessary, an appropriate transformation was used. Likelihood ratio tests (LRTs) were used to compare the full model, with models excluding each fixed or interaction term of interest. This allowed us to determine the significance of the term by measuring the deviance from the full model, using LRT χ² tests. Interaction effects are presented regardless of their significance. Effect sizes of main and interaction effects were measured using partial *η*^2^ and are interpreted as negligible if less than 0.01; small if between 0.01 and 0.06; moderate if between 0.06 and 0.14; and large if greater than 0.14 [47]. The *emmeans* package allowed us to extract least square means (presented in the text together with standard error) for each variable. Planned or *post hoc* pairwise comparisons were used to measure differences between groups. Where possible (i.e. we had specific hypotheses and predictions), we minimized the number of planned pairwise comparisons made and used a *p*-value adjustment for multiple comparisons following the Holm method for the relevant number of tests. Effect sizes for the paired differences were calculated as Cohen’s *d* based on the least square means generated from each model and are therefore only relevant in the context of each model. Effect sizes were considered small if Cohen’s *d* was less than 0.5, medium if between 0.5 and 0.8 and large if greater than 0.8 [47].

## 3. Results

***Aim 1:** to replicate previous results highlighting environmental and genetic aetiological factors of inactive but awake behaviour (pre-environmental adjustment data only).*

**Figure 3.**
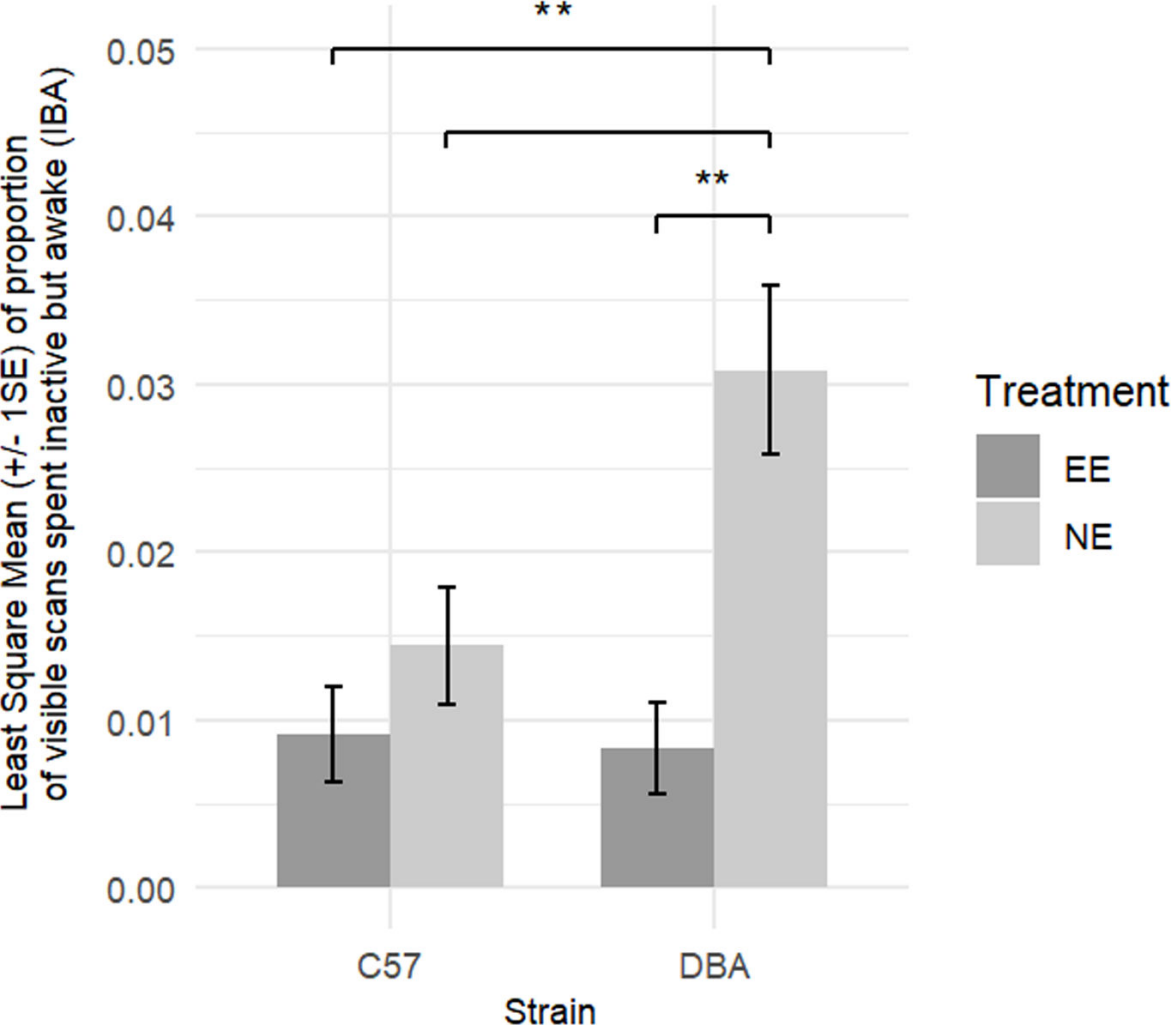
Least square means (±1 s.e.) of proportion of visible scans in which mice were inactive but awake (IBA) during the three-week period before environmental adjustment (IBA observation 1), split by strain (C57: C57BL/6J, DBA: DBA/2J). During this pre-environmental adjustment phase, all mice were housed in either EE (dark grey bars) or relatively NE (light grey bars) cages. *n*_NE DBAs_ = 16 mice, *n*_NE C57s_ = 16 mice, *n*_EE DBAs_ = 15 mice, *n*_EE C57s_ = 15 mice. Overall, DBA mice spent a greater proportion of their visible time performing IBA behaviour than C57 mice, and did so more in the NE compared with EE cages (EE (*c*) versus NE (DBA): *p* = 0.0029; NE (*c*) versus NE (DBA): *p* = 0.0137; EE (DBA) versus NE (DBA): *p* = 0.0019).

Based on previous results from [2] and [15], we predicted that NE cages would trigger higher levels of IBA behaviour, compared with EE cages. We also expected to see a significant genetic component to the development of IBA and that such a strain-related difference would be exacerbated in NE cages, although previous work has shown varied results (C57 mice displayed more IBA than DBAs in [2] and [15] and no significant strain effect in [3]). As expected, mice spent a greater proportion of their visible scans displaying IBA behaviour in the NE than in the EE cages before environmental adjustment (NE_*n* = 32 mice_ = 0.022 ± 0.003; EE_*n* = 30 mice_ = 0.009 ± 0.002; planned contrasts: *t*_29_ = 3.303, *p* = 0.003, Cohen’s *d* = 0.814). Strains also differed in the time they spent displaying IBA behaviour, although in the current study, DBA mice (as compared with C57 mice) tended to show a greater proportion of visible scans displaying IBA behaviour (DBAs_*n* = 31_ = 0.018 ± 0.003; C57s_*n* = 31_ = 0.012 ± 0.002, planned contrasts: *t*_29_ = 2.037, *p* = 0.051, Cohen’s *d* = 0.444). Such strain-related differences were exacerbated when the mice were housed in NE cages (strain × environment interaction LRT χ²_1_ = 5.604, *p* = 0.018, partial *η*^2^ = 0.165), with NE DBA mice showing significantly more IBA behaviour than mice in all other conditions (planned contrasts: NE DBAs versus EE C57s: *t*_29_ = 3.862, *p* = 0.003, Cohen’s *d* = 1.320; NE DBAs versus NE C57s: *t*_29_ = 3.187, *p* = 0.014, Cohen’s *d* = 0.950; NE DBAs versus EE DBAs: t_29_ = 4.080, *p* = 0.002, Cohen’s *d* = 1.385; *p* > 0.1 for other pairwise comparisons, all Cohen’s *d* < 0.5; figure 3).

***Aim 2:** to test whether the amount of time mice spent displaying IBA behaviour would increase with environmental enrichment reduction, and (conversely) would decrease when provided with environmental enrichment enhancement (by measuring change in IBA behaviour pre-/post-environmental adjustment).*

**Figure 4.**
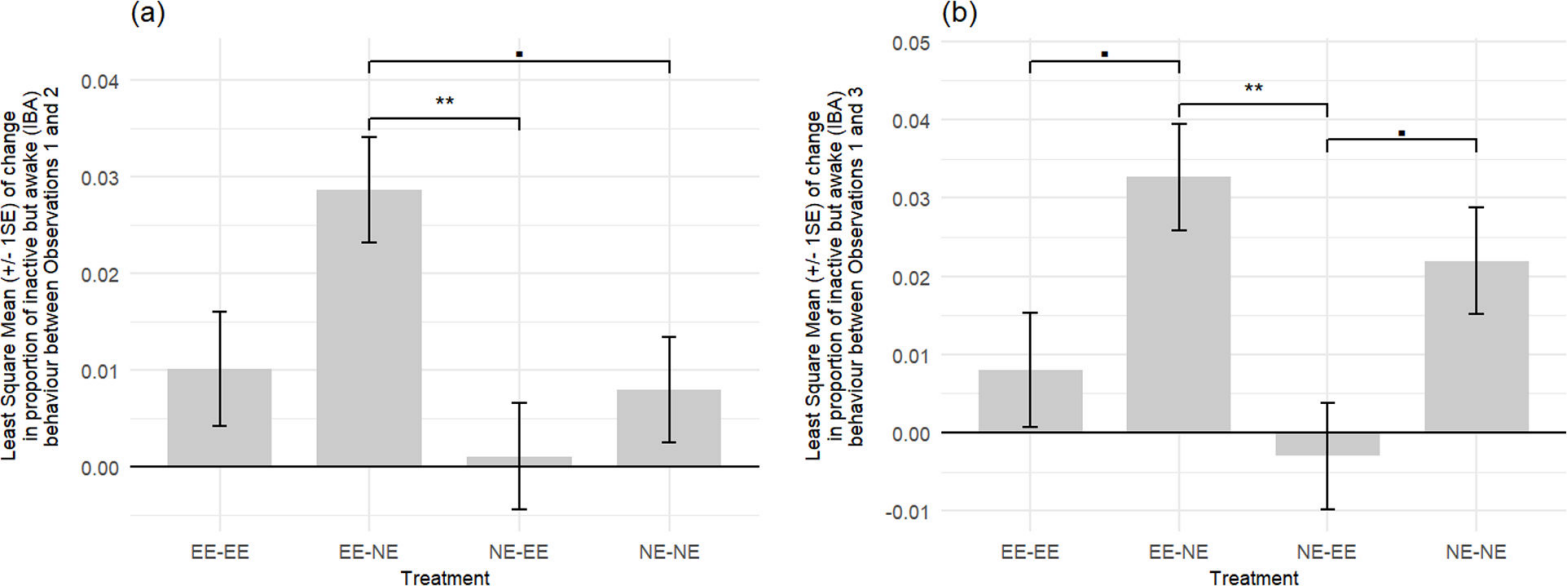
Least square means of change (±1 s.e.) in the proportion of visible scans in which mice were inactive but awake (IBA) (a) comparing IBA levels during the three weeks of housing before the environmental adjustment, with the three weeks of housing post-environmental adjustment to IBA levels (referred to in the axis as between observations 1 and 2). EE-NE versus NE-NE: *p* = 0.0629; EE-NE versus NE-EE: *p* = 0.0084. (b) Comparing IBA levels during the three weeks of housing before the environmental adjustment, with IBA levels on the eighth week post-environmental adjustment (referred to in the axis as between observations 1 and 3). None of the groups differed from each other in their change in proportions of visible scans displaying IBA behaviour between observations 2 and 3 (as presented in table 1). Group EE-EE: mice were initially housed in EE cages and stayed in this initial environment (control group, *n* = 8 cages /16 mice); group EE-NE: mice were initially housed in EE cages and moved to NE cages (*n* = 7 cages /14 mice); group NE-EE: mice were initially housed in NE cages and moved to EE cages (*n* = 8 cages /16 mice); group NE-NE: mice were initially housed in NE cages and stayed in this initial environment (control group, *n* = 8 cages /16 mice). Positive values indicate that the proportion of visible scans in which mice were displaying IBA behaviour increases between observations, and negative values indicate a decrease in IBA levels between observations. EE-EE versus EE-NE: *p* = 0.0780; EE-NE versus NE-EE: *p* = 0.0055; NE-EE versus NE-NE: *p* = 0.0744.

**Figure 5.**
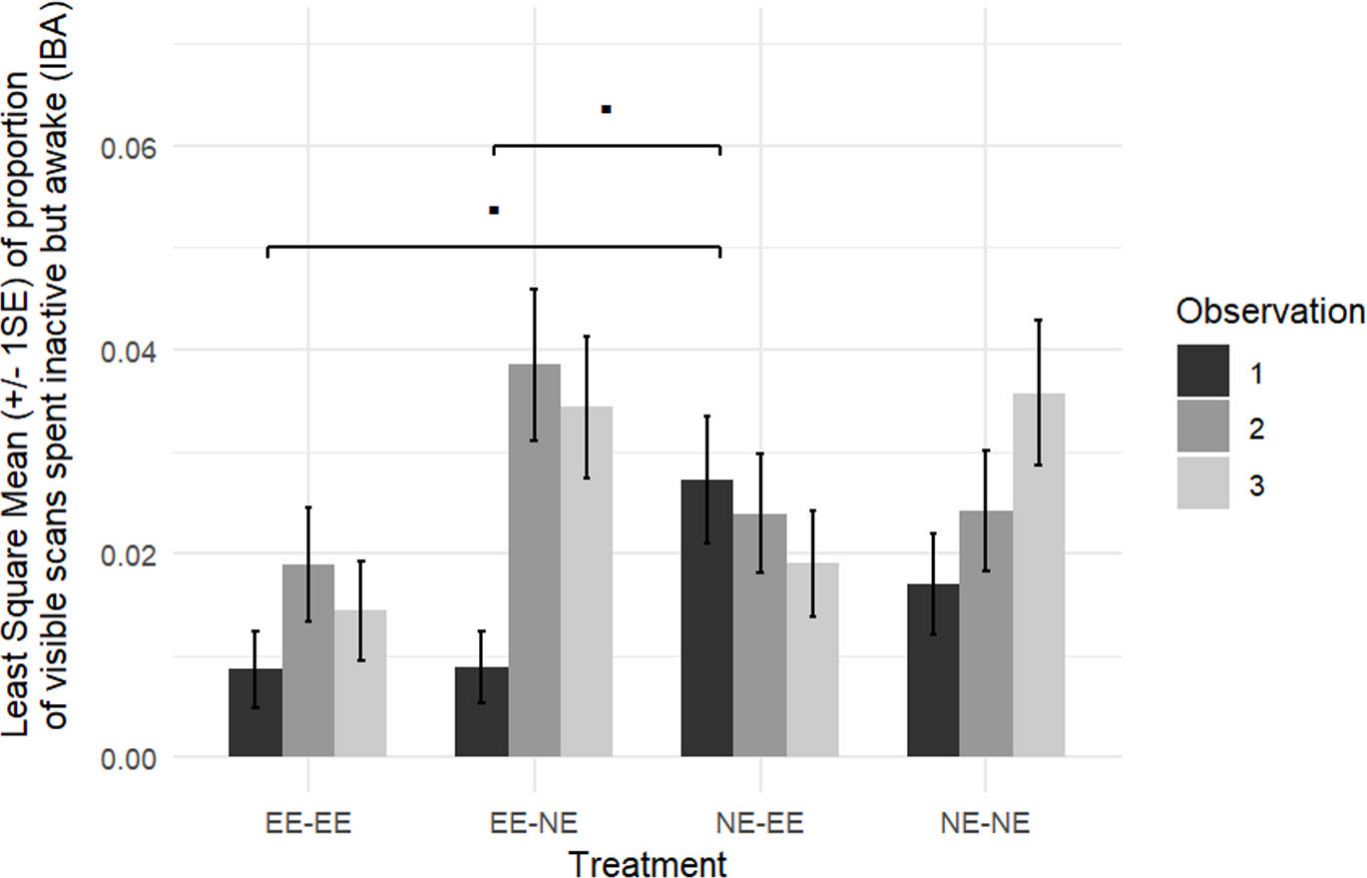
Least square means of change (±1 s.e.) in the proportion of visible scans in which mice were inactive but awake (IBA) comparing IBA levels during the three weeks of housing before the environmental adjustment (observation 1), with the three weeks of housing post-environmental adjustment (observation 2) and on the eighth week post-environmental adjustment (observation 3). Treatment groups are as defined in figure 4. During observation 1, there tended to be more IBA behaviour shown by the NE-EE group than mice in the EE-EE (*p* = 0.0769) and EE-NE (*p* = 0.0769) groups.

The proportion of visible scans in which mice displayed IBA behaviour in the two control groups housed in stable conditions throughout the experiment (NE for group NE-NE mice and EE for group EE-EE mice) increased similarly over time in both groups (NE-NE versus EE-EE: observations 1–2, *t*_27_ = 0.271, *p* = 0.809, Cohen’s *d* = 0.090; observations 1–3, *t*_27_ = 1.403, *p* = 0.516, Cohen’s *d* = 0.514) (table 1, figures 4 and 5).

**Table 1.** *Post hoc* contrasts to compare the change in the proportion of visible scans during which mice displayed inactive but awake (IBA) between the three observations for different treatment groups (see also figure 5). *p*-value adjustment: Holm method for six tests. Trends (*p* < 0.1) are presented in italics and significant differences (*p* < 0.05) are presented in bold. EE-EE: mice were initially housed in enriched cages and stayed in this initial environment throughout the experiment (control group, *n* = 7 cages / 14 mice); EE-NE: mice were initially housed in enriched cages and moved to non-enriched cages (*n* = 8 cages / 16 mice); NE-EE: mice were initially housed in non-enriched cages and moved to enriched cages (*n* = 8 cages / 16 mice); NE-NE: mice were initially housed in non-enriched cages and stayed in this initial environment throughout the experiment (control group, *n* = 8 cages / 16 mice). IBA observation 1: three weeks prior to environmental adjustment; IBA observation 2: three weeks following environmental adjustment; IBA observation 3: the eighth week following environmental adjustment. Overall, exposing mice to a reduction in home-cage environmental enrichment (group EE-NE) induces a greater increase in IBA behaviour, or tends to, compared with housing mice in enriched cages throughout (group EE-EE), to housing mice in non-enriched cages throughout (group NE-NE) and to exposing mice to an increase in environmental enrichment (positive contrast group NE-EE), while the latter positive contrast appears to reduce the amount of IBA behaviour mice display compared with mice remaining in non-enriched cages throughout (group NE-NE). Although the absolute levels of IBA behaviour in NE cages was lower than in EE cages, the results in table 2 and figure 5 display the change in levels of IBA behaviour between observations, hence we did not observe differences between the groups which remained stable (NE-NE and EE-EE).

observation contrast	treatment contrast	estimate	s.e.	*t* ratio	*p*-value
observations 1–2	NE-EE versus NE-NE	−0.00687	0.00775	−0.887	0.8088
	*EE-NE versus NE-NE*	*0.02073*	*0.00775*	*2.673*	*0.0629*
	**EE-NE versus NE-EE**	**0.02760**	**0.00775**	**3.560**	**0.0084**
	EE-NE versus EE-EE	0.01856	0.00803	2.312	0.1145
	EE-EE versus NE-NE	0.00217	0.00803	0.271	0.8088
	EE-EE versus NE-EE	0.00905	0.00803	1.127	0.8088
observations 1–3	*NE-EE versus NE-NE*	*−0.0249*	*0.00959*	*−2.601*	*0.0744*
	EE-NE versus NE-NE	0.0107	0.00959	1.118	0.5466
	**EE-NE versus NE-EE**	**0.0357**	**0.00959**	**3.720**	**0.0055**
	*EE-NE versus EE-EE*	*0.0246*	*0.00992*	*2.484*	*0.0780*
	EE-EE versus NE-NE	−0.0139	0.00992	−1.403	0.5158
	EE-EE versus NE-EE	0.0110	0.00992	1.110	0.5466
observations 2–3	NE-EE versus NE-NE	−0.01806	0.0118	−1.532	0.8230
	EE-NE versus NE-NE	−0.01001	0.0118	−0.849	1.0000
	EE-NE versus NE-EE	0.00806	0.0118	0.683	1.0000
	EE-NE versus EE-EE	−0.00609	0.0122	−0.499	1.0000
	EE-EE versus NE-NE	−0.01610	0.0122	−1.319	0.9916
	EE-EE versus NE-EE	0.00197	0.0122	0.161	1.0000

When considering all four groups, we found (as expected) a significant effect of treatment (LRT observation 1–2, χ²_3_ = 12.853, *p* = 0.005, partial *η*^2^ = 0.339; LRT observation 1–3, χ²_3_ = 14.987, *p* = 0.002, partial *η*^2^ = 0.369). As predicted, the mice that had been exposed to a reduction in home-cage environmental enrichment (group EE-NE) tended to show a greater increase in IBA by observation 3 compared with the control mice that remained in EE cages throughout the experiment (group EE-EE) (*t*_27_ = 2.484, *p* = 0.078, Cohen’s *d* = 0.909). This difference was not significant for IBA observation 2, although a large effect size was generated (*t*_27_ = 2.312, *p* = 0.115, Cohen’s *d* = 0.846) (table 1, figure 4). The group EE-NE mice also tended to show a greater increase in IBA behaviour between observations 1 and 2 than the stably housed, group NE-NE mice (housed in NE cages throughout the experiment) (*t*_27_ = 2.673, *p* = 0.063, Cohen’s *d* = 0.945). This difference was, however, not significant between observations 1 and 3 (*t*_27_ = 1.118, *p* = 0.547, Cohen’s *d* = 0.395). When compared with the group NE-EE mice exposed to an *increase* in environmental enrichment, the group EE-NE mice showed a greater increase in IBA between observations 1 and 2 (*t*_27_ = 3.560, *p* = 0.008, Cohen’s *d* = 1.259, table 1, figure 4) and observations 1 and 3 (*t*_27_ = 3.720, *p* = 0.006, Cohen’s *d* = 1.315, table 1, figure 4).

Furthermore, and as predicted, the control group NE-NE mice (remaining in NE cages throughout) tended to show a greater increase in IBA behaviour by observation 3, when compared with the group NE-EE mice that had been exposed to an *increase* in environmental enrichment (which generally showed a *reduction* in IBA behaviour) (*t*_27_ = 2.601, *p* = 0.074, Cohen’s *d* = 0.920, table 1, figure 4). This effect, however, did not reach significance for observation 2 (*t*_27_ = 0.887, *p* = 0.809, Cohen’s *d* = 0.312, table 1, figure 4).

We found no significant interaction between treatment × strain between observations 1 and 2 (LRT χ²_3_ = 1.103, *p* = 0.776, partial *η*^2^ = 0.035) or between observations 1 and 3 (LRT χ²_3_ = 0.053, *p* = 0.997, partial *η*^2^ = 0.002).

***Aim 3:** to investigate whether greater levels of inactive but awake predict lower immature neuron density in the dorsal or ventral dentate gyrus.*

We expected that greater levels of IBA behaviour would predict reduced immature neuron density in the dorsal and ventral dentate gyrus region of the hippocampus. Specifically, we predicted that mice housed in the EE cages for the duration of the study (i.e. EE-EE group) would display less IBA behaviour (compared with group NE-NE) and have higher immature neuron densities.

Overall, we found significant treatment × region (LRT χ²_1_ = 11.338, *p* < 0.001, partial *η*^2^ = 0.125) and strain × region (LRT χ^²^_1_ = 57.332, *p* < 0.001, partial *η*^2^ = 0.656) interaction effects. *Post hoc* contrasts revealed that significantly greater densities were measured in the dDG compared with the vDG (dDG: 0.079 ± 0.005; vDG: 0.026 ± 0.003; *t*_25_ = 15.051, *p* < 0.001, Cohen’s *d* = 2.568) and this was true for both treatment groups. Between treatment groups, the EE-EE group had a significantly greater immature neuron density in the vDG compared with the NE-NE group (EE-EE: 0.036 ± 0.004; NE-NE: 0.017 ± 0.003; *t*_12_ = 3.465, *p* = 0.009, Cohen’s *d* = 1.303). However, there was no significant treatment-related difference in the immature neuron density in the dDG (*t*_12_ = 0.284, *p* = 0.781, Cohen’s *d* = 0.107).

**Figure 6.**
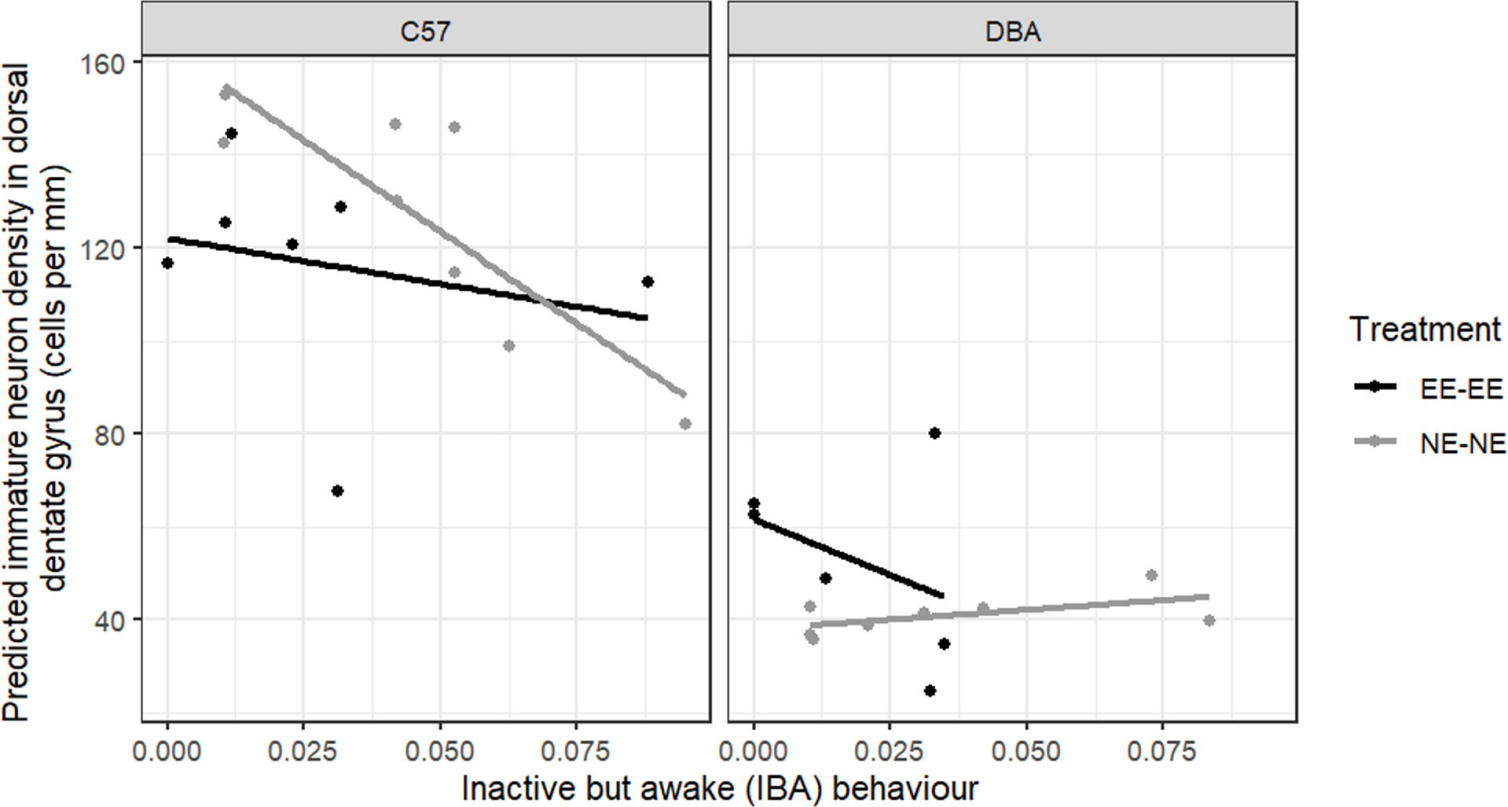
Predicted immature neuron density in the dDG (cells per mm), as predicted by IBA behaviour displayed during the eighth week following environmental adjustment (observation 3), for the EE-EE (stably housed in EE cages: black lines and points) and NE-NE (stably housed in NE cages: grey lines and points) treatment groups. Individual points represent data for each mouse. The figure is split by strain of mice (C57: C57BL/6J, DBA: DBA/2J). *n*_NE-NE DBAs_ = 8 mice, *n*_NE-NE C57s_ = 8 mice, *n*_EE-EE DBAs_ = 6 mice, *n*_EE-EE C57s_ = 7 mice. Overall, C57 mice had a greater immature neuron density in the dDG than DBA mice, even more so in group NE-NE as opposed to group EE-EE (*p* < 0.0001). Greater levels of IBA predicted reduced immature neuron density in the dDG (χ²_1_ = 4.101, *p* = 0.0429), and tended to do so even more for C57 mice (strain × IBA interaction: *p* = 0.0510).

**Figure 7.**
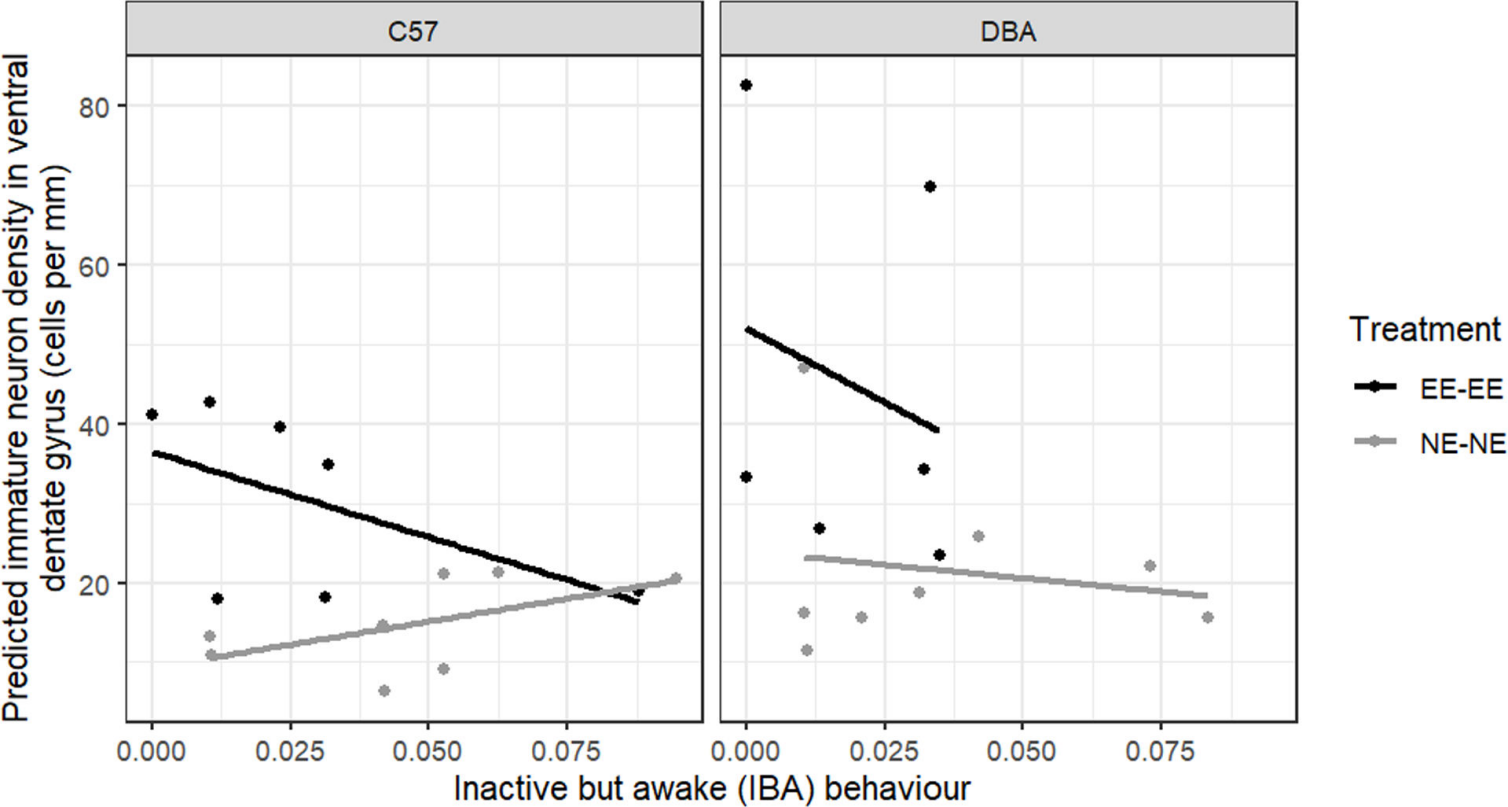
Predicted immature neuron density in the vDG (cells per mm), as predicted by IBA behaviour displayed during the eighth week following environmental adjustment (observation 3), for the EE-EE (stably housed in EE cages: black lines and points) and NE-NE (stably housed in NE cages: grey lines and points) treatment groups. Individual points represent data for each mouse. The figure is split by strain of mice (C57: C57BL/6J, DBA: DBA/2J). *n*_NE-NE DBAs_ = 8 mice, *n*_NE-NE C57s_ = 8 mice, *n*_EE-EE DBAs_ = 6 mice, *n*_EE-EE C57s_ = 7 mice. IBA levels did not predict immature neuron density in the vDG. Overall, DBA mice had a greater immature neuron density in the vDG than did C57 mice (*p* = 0.0499). Greater immature neuron densities were measured in mice in the EE-EE group compared with the NE-NE group (*p* = 0.00478).

When investigating the effect of IBA on the immature neuron density in the dDG, we found significant strain (LRT χ²_1_ = 48.620, *p* < 0.001, partial *η*^2^ = 0.963) and IBA behaviour (LRT χ²_1_ = 4.101, *p* = 0.043, partial *η*^2^ = 0.378) main effects, with being a DBA mouse, and having a higher level of IBA behaviour both predicting reduced immature neuron density in the dDG. Furthermore, we found a strain × IBA interaction trend (LRT χ²_1_ = 3.808, *p* = 0.051, partial *η*^2^ = 0.284, figure 6), with greater levels of IBA predicting reduced dDG immature neuron density even more so for C57 mice (figure 7). We also found a significant treatment × strain interaction (LRT χ²_1_ = 7.890, *p* = 0.005, partial *η*^2^ = 0.344, figure 6). *Post hoc* comparisons revealed a stark difference in immature neuron density between the two strains of mice, with C57 mice having a higher density than DBA mice (C57_*n* = 15_: 0.126 ± 0.005; DBA_*n* = 14_: 0.045 ± 0.006, Cohen’s *d* = 3.718; main effect of strain: χ²_1_ = 48.618, *p* < 0.001, partial *η*^2^ = 0.963), and even more so for group NE-NE (C57_*n* = 8_: 0.136 ± 0.007; DBA_*n* = 8_: 0.041 ± 0.007; *t*_8_ = 12.493, *p* < 0.0001, Cohen’s *d* = 4.723, figure 6).

When investigating the effect of IBA behaviour on the immature neuron density in the vDG, we found significant main effects of both treatment (LRT χ²_1_ = 7.961, *p* = 0.005, partial *η*^2^ = 0.372, figure 7) and strain (χ²_1_ = 3.846, *p* = 0.050, partial *η*^2^ = 0.366, figure 7), but no significant interaction effects. *Post hoc* contrasts confirmed that overall greater cell densities were measured in mice in the EE-EE group (EE-EE_*n* = 13_: 0.036 ± 0.006; NE-NE_*n* = 16_: 0.017 ± 0.005, Cohen’s *d* = 0.928). Interestingly, unlike the dDG, overall immature neuron density in the vDG was greater for DBA than C57 mice (DBA_*n* = 14_: 0.032 ± 0.005; C57 _*n* = 15_: 0.021 ± 0.004, Cohen’s *d* = 0.587). There was no significant main effect of IBA behaviour on vDG immature neuron density (LRT χ²_1_ = 0.003, *p* = 0.956, partial *η*^2^ < 0.001) and no significant IBA*treatment or IBA*strain interaction effects.

## 4. Discussion

In relation to our first aim, we found, as expected, that mice spent a greater proportion of their visible time displaying IBA in the NE than in the EE cages before the environmental adjustment, with a large effect size. We also expected that there would be a genetic influence on the tendency to perform IBA, although previous results are conflicting (more in C57 [2,15]; no significant strain difference [3]). In fact, we found that DBAs tended to show greater levels of IBA than C57s (i.e. opposite finding to [2], particularly when housed in NE cages. The inconsistency in strain differences across experiments is perhaps unsurprising [48], given that the mice were obtained from different breeding laboratories (we used mice from Charles River in France, while previous work [2,15] used mice from Charles River in North America). This highlights the need for greater acknowledgement that differences in laboratory rearing conditions and differences between genetic lines may have a profound influence on the development of behavioural conditions (e.g. [48]). In fact, it has been suggested that introducing systematic heterogenization (e.g. different strains or sub-strains) into biological experimental design could hugely improve the external validity of research models [48–50]. Additional (and not mutually exclusive) factors to account for the behavioural differences observed between strains here and in the aforementioned studies are variation in the age of the mice at the start of experiments, and different local laboratory environments (e.g. [22]).

The main results from our first aim affirm that both environment and genetics can play a role in IBA behaviour development, as also suggested by the diathesis-stress model of depression in humans [20]. In the current study, environmental factors had a larger effect size on the performance of IBA behaviour than genetics alone. Similarly in humans, environmental factors may have a larger influence over the development of depression than inherited traits [51] and depression is only moderately heritable (range 30–50%) [52,53]. In our other research, we found that increasing environmental enrichment alone was as effective (and even had a greater effect size, unpublished data) as an antidepressant drug (chronic Venlafaxine) at reducing IBA triggered by NE cages [3]. Additionally, in this work, the provision of an antidepressant drug alone was not sufficient in preventing the rise in IBA generated by enrichment loss, further indicating the relative importance of environmental factors in the development of depression-like symptoms in mice.

To address our second aim, two groups of mice underwent an environmental adjustment to their housing condition. The other two groups remained stably housed in either the EE cages or NE cages. Although mice in the NE cages showed greater levels of IBA behaviour throughout the experiment, the two stably housed groups of mice did not differ in the magnitude of *change* in IBA behaviour. This suggests that even though lower levels were observed in the stably housed enriched condition throughout, IBA behaviour was still displayed to a degree by some mice, hence the enrichment was not sufficient in preventing *all* cases of IBA behaviour, as had been displayed in previous work [2,3,15]. This may be because, although the EE condition is more refined than standard laboratory cage housing, it is still a long way from fully mimicking a natural environment for mice [54,55]. For instance, some mice housed in highly EE cages still display stereotypic behaviours (e.g. [2]). An even more complex, EE and naturalistic environment may be required to fully alleviate such signs of poor welfare, although the feasibility of this approach in a laboratory setting may be challenging.

For the mice that had enrichment removed (group EE-NE), compared with those stably housed in a NE condition (group NE-NE), a sharp increase in IBA behaviour was shown, as expected, indicating they were adjusting their behaviour in response to the environment within three weeks. Furthermore, the mice that had enrichment *removed* displayed significantly more IBA behaviour both three and eight weeks after environmental adjustment (IBA observations 2 and 3) than the mice that *gained* enrichment. These two results support our prediction that the enrichment loss/gain would mediate IBA behaviour levels. When compared with the stably housed EE mice (group EE-EE), those which had enrichment removed (group EE-NE) tended to show a greater increase in IBA behaviour eight weeks after enrichment removal (IBA observation 3), although this was not apparent during the three weeks following enrichment removal (IBA observation 2). As suggested by the large error bars and visual inspection of our data**,** IBA behaviour appears to be displayed more frequently by some mice than others and as discussed above, even in the EE environment by a few mice—see figure 5. This could have resulted in the lack of a significant short-term difference seen here. Although it sounds counter-intuitive, this inter-individual variability may further support IBA behaviour as a candidate indicator of a depression-like state in mice. Indeed, due to the complex aetiology of mental disorders, we would not expect *every* individual to develop either depression-like features or elevated IBA behaviour as a result of the environmental conditions [56].

We also found that, as predicted, mice that had been exposed to an increase in environmental enrichment generally showed a *reduction* in IBA behaviour. Conversely, mice remaining in the NE condition tended to show a greater increase in IBA behaviour eight weeks later (IBA observation 3). The difference between these two treatment groups was, however, not yet significant after just three weeks (IBA observation 2). This delay between enrichment provision and a change in behaviour is potentially understandable, particularly if IBA behaviour had become instilled in the behavioural repertoire of some mice (i.e. habitual), while their affective state may have, in fact, improved. Human literature suggests that it can take over two months to change a behavioural sign of depression, even when reported depressive feelings have abated (depending on the behaviour) [57], so it is possible that there may also be a delay for non-human animals. Furthermore, as with human depression, curative factors are unlikely to be instantaneous and often take time to be effective, both for cognitive/behavioural changes and pharmacological therapies. Alternatively, in other work, we found a significant difference between similar treatment groups three weeks after enrichment removal [3]. Hence, further research on the timing of the development of IBA is required.

Our third aim was to opportunistically investigate whether greater levels of IBA behaviour predict reduced immature neuron density in the hippocampus. Immature neuron density was significantly greater in the dDG than the vDG, which is in accordance with previous work finding that neurons in the vDG mature more slowly [34,35]. As expected, in vDG, we measured lower overall immature neuron density in the NE, NE cages compared with the EE cages [56,58], in both strains. We did not pick up this main effect of enrichment in the dDG, which is surprising, as most previous studies have only looked at dDG and found effects of enrichment there [58,59]. In vDG, DBA mice had significantly greater immature neuron densities than the C57s, while in the dDG, DBA mice had fewer immature neurons than C57 mice. This is an interesting finding, as Kim et al. [60] concluded that DBA/2 mice were not useful for studying adult hippocampal neurogenesis, due to their low levels of neurogenesis, but they only studied the dorsal hippocampus. Strain differences in immature neuron densities in dDG and vDG might relate to differences in cognitive abilities and stress resilience, respectively [61,62].

Greater levels of IBA behaviour predicted reduced immature neuron density in the dDG, and this effect tended to be stronger for C57 mice. This result is consistent with former observations that C57 mice displaying the greatest levels of IBA behaviour had reduced hippocampal volume [19]. Moreover, C57 females that showed greater inactivity in the forced swim test had fewer immature neurons in the dDG [63], further suggesting a link between reduced dDG neurogenesis and behavioural signs of depression in mice (although we acknowledge the debate surrounding the interpretation of the forced swim test—e.g. [64–66]). In the mammalian hippocampus, the dDG (termed posterior DG in primates) is thought to be involved in some memory processing [67]. Accordingly, in some humans with clinical depression, hippocampal neurogenesis is reduced [26–28] and the frequency of self-reported memory problems increases with greater scores on the hospital anxiety and depression scale [68]. Hence, our finding that greater levels of IBA behaviour predicted reduced dDG immature neuron density is consistent with the hypothesis that greater IBA behaviour may reflect a depression-like state in mice. The fact that this relationship did not exist in vDG may be due to a floor effect in the vDG: if vDG is more sensitive to negative environmental effects (as evidenced by the treatment × region interaction effect), it will respond maximally to these in all animals leading to low levels of neurogenesis and correspondingly low (floor) levels of immature neurons across all subjects. However, dDG, being less sensitive, will respond to negative environmental effects in a non-maximal way that allows the effects of individual differences in depression-like state (as indicated by IBA) to be observed.

## 5. Conclusions

What is the future direction for IBA behaviour research? These initial results warrant further investigation. To further validate IBA behaviour from a behavioural perspective, we need to investigate the covariation of the frequency of IBA behaviour within the same individual mice with a *range* of diagnostic criteria and features of human depression [3,13]. Indeed, depression is a heterogeneous condition and its diagnosis in humans requires that at least five of the nine symptoms is displayed at least over a period of two weeks and represent a change from previous functioning [18]. Moreover, the temporal development of IBA behaviour (e.g. duration of episode, age of onset) has not yet been investigated and this is important because depression is a complex, commonly recurrent and sometimes long-lasting disorder in humans [18]. Furthermore, thus far we have focused only on female mice because depression is more prevalent in women. However, elevated IBA states are unlikely to be sex-specific and also warrant investigation in male mice, especially since no clear sex differences exist in symptoms, course, treatment response or functional consequences in human depression [18].

From a biomarker perspective, we measured immature neuron density in the hippocampi of the mice opportunistically, with no opportunity to develop a power calculation. These results are therefore exploratory, and replication with a pre-calculated sample size would be needed to make further conclusions about the link between IBA behaviour and DG immature neuron density. However, the results here show that IBA behaviour has promise as a non-invasive measure of mouse welfare that could be used across a range of experiments. Additionally, IBA behaviour could serve as a novel read-out for preclinical models of depression which can be observed in the home cage, reducing the need for invasive tests such as the controversial forced swim test [64–66]. Furthermore, investigating the covariation of IBA behaviour with a range of other potential biomarkers of depression, such as hormones (e.g. cortisol [69]) and inflammatory biomarkers (e.g. cytokines [45]) would be useful.

Despite the need for further research, the results presented here provide evidence that conventional laboratory housing is not sufficiently enriched to alleviate behavioural and neuronal abnormalities. Aligned with the 3Rs refinement recommendations, additional research into the uptake and implementation of complex enrichment into laboratory housing for mice would be valuable.

## Data Availability

All data and R script files used for statistical analyses and figure generation can be accessed in the Dryad repository [70].
